# High glucose uptake unexpectedly is accompanied by high levels of the mitochondrial β-F1-ATPase subunit in head and neck squamous cell carcinoma

**DOI:** 10.18632/oncotarget.5459

**Published:** 2015-10-06

**Authors:** Christian U. Huebbers, Alexander C. Adam, Simon F. Preuss, Theresa Schiffer, Sarah Schilder, Orlando Guntinas-Lichius, Matthias Schmidt, Jens P. Klussmann, Rudolf J. Wiesner

**Affiliations:** ^1^ Jean-Uhrmacher-Institute for Otorhinolaryngological Research, University of Köln, 50924 Köln, Germany; ^2^ Department of Pathology, Medical Faculty, University of Köln, 50924 Köln, Germany; ^3^ Department of Otolaryngology, Medical Faculty, University of Köln, 50924 Köln, Germany; ^4^ Center for Physiology and Pathophysiology, Institute of Vegetative Physiology, Medical Faculty, University of Köln, 50931 Köln, Germany; ^5^ Department of Otorhinolaryngology, Jena University Hospital, 07740 Jena, Germany; ^6^ Department of Nuclear Medicine, Medical Faculty, University of Köln, 50924 Köln, Germany; ^7^ Department of Otorhinolaryngology, Head and Neck Surgery, University of Giessen, 35385 Giessen, Germany; ^8^ Center for Molecular Medicine Cologne, CMMC, University of Köln, 50931 Köln, Germany; ^9^ Cologne Excellence Cluster on Cellular Stress Responses in Ageing-associated Diseases (CECAD), 50674 Köln, Germany

**Keywords:** HNSCC, OXPHOS, ^18^F-FDG-PET, Warburg effect

## Abstract

A hallmark of solid tumors is the consumption of large amounts of glucose and production of lactate, also known as Warburg-like metabolism. This metabolic phenotype is typical for aggressive tumor growth, and can be visualized by ^18^F-fluorodeoxyglucose (^18^F-FDG) uptake detected by positron emission tomography (PET). High ^18^F-FDG uptake inversely correlates with survival and goes along with reduced expression of the catalytic beta-subunit of the H^+^-ATP synthase (β-F1-ATPase) in several tumor entities analyzed so far.

For this study we characterized a series of 15 head and neck squamous cell carcinoma (HNSCC) by (i) determining ^18^F-FDG-uptake; (ii) quantitative expression analysis of β-F1-ATPase (Complex V), NDUF-S1 (Complex I) and COX1 (Complex IV) of the mitochondrial electron transport chain (ETC), as well as Hsp60 (mitochondrial mass) and GAPDH (glycolysis) in tumor cells; (iii) sequencing of the mtDNA of representative tumor samples.

Whereas high ^18^F-FDG-uptake also correlates with poor prognosis in HNSCC, it surprisingly is accompanied by high levels of β-F1-ATPase, but not by any of the other analyzed proteins.

In conclusion, we here describe a completely new phenotype of metabolic adaptation possibly enabling those tumors with highest levels of β-F1-ATPase to rapidly proliferate even in hypoxic zones, which are typical for HNSCC.

## INTRODUCTION

Solid tumors consume large amounts of glucose, a property which is used by ^18^F-fluorodeoxyglucose positron emission tomography (^18^F-FDG-PET) for detection as well as quantification of tumor size and growth, e.g. in order to monitor success of treatment regimens. ^18^F-FDG uptake has been shown to inversely correlate with survival, which indicates that a high glycolytic rate is typical for aggressive tumor growth [1–3]. Tumors also produce large amounts of lactate, and these two phenomena were proposed to be a hallmark of cancer by Otto Warburg [4]. He postulated that this metabolic phenotype was necessary for ATP production in order to survive in the often hypoxic tumor micro-environment and was similar to anaerobic glycolysis in underperfused tissue, e.g. muscle upon onset of exercise. However, a Warburg-like metabolism, then termed aerobic glycolysis, can be observed (i) even if a solid tumor is well perfused, (ii) in leukemic cells in the bloodstream and (iii) in most cultured tumor cells routinely grown under even hyperoxic conditions, showing that it is a fixed part of the tumor program (reviewed in [5]). Uptake of glucose and conversion to lactate in the presence of oxygen seems to be a waste of precious reduced carbon, even for “selfish” tumor cells. This metabolic pathway not only produces 18-times less ATP compared to complete oxidation by mitochondrial oxidative phosphorylation (OXPHOS), but also leads to loss of the reduced carbon taken up before, which in turn is the source of building blocks needed for cell proliferation. Therefore, Warburg-like metabolism, as it is seen today, includes the simultaneous conversion of part of the consumed glucose to ribose-5-phosphate for nucleotide synthesis (pentose-phosphate-shunt). This simultaneously generates NADPH as necessary reducing power for membrane lipid synthesis. At the same time, large amounts of glutamine are converted to mitochondrial tricarboxylic acid (TCA) cycle intermediates in most tumors, providing the nitrogen for nucleotide synthesis. In addition, this “anaplerosis” also compensates for the export of mitochondrial citrate to the cytosol, where this metabolite is used to generate acetyl-CoA, again used for membrane lipid synthesis. Labeling studies have shown that acetyl-CoA for lipid synthesis is ultimately derived from glucose. In addition, a considerable amount of the lactate secreted by tumor cells is not derived from glucose, but from glutamine via the TCA cycle and cytosolic malic enzyme [6], reviewed in [5]. In summary, it is the need for large amounts of carbon and nitrogen as building blocks for membrane lipids, nucleotides and proteins as well as the NADPH-reducing power necessary for their synthesis, and not a high energy demand, which drives high rates of glucose (and glutamine) uptake of rapidly proliferating tumor cells. Lactate production from pyruvate, which is either derived from glucose or from glutamine, is now rather thought to assist in the maintenance of a proper redox balance of the NADH/NAD couple, allowing conversion of large amounts of glucose to acetyl-CoA as a precursor for lipid synthesis [7].

Warburg had initially postulated that “aerobic glycolysis” is due to defects of the mitochondrial electron transport chain (ETC), which usually converts reducing power derived from glucose and lipids into water and ATP, but this has been questioned already at Warburg's time and still is (for review, see [5, 8]). However, although it is now clear that tumor cells do have active mitochondria capable of respiring [9], a large body of data shows that Warburg's original postulate is still true to some extent for many tumor entities: Especially Cuezva and coworkers have demonstrated, carefully quantitating immunohistochemical staining of tumor cells in various tumors, that especially the expression of the β-subunit of complex V (β-F1-ATPase), the mitochondrial H^+^-ATP synthase, is significantly reduced [10]. They dubbed a bioenergetic cellular index (BEC-index: levels of β-F1-ATPase/Hsp60/GAPDH) [11, 12] and showed that this value inversely correlated with ^18^F-FDG-uptake in lung tumors [13]. Thus, they demonstrated that low H^+^-ATP synthase levels relative to mitochondrial mass (Hsp60) and to glycolytic capacity (GAPDH), respectively, may serve as a new predictor for malignancy and patient survival, at least for these tumors. Recently, they have shown that in addition, high levels of the endogenous peptide inhibitor of the mitochondrial H^+^-ATP synthase, ATPase inhibitory factor 1 (IF1), are present in colon, lung, breast and ovarian carcinomas, possibly being another mechanism to inhibit still remaining H^+^-ATP synthase activity [10, 14].

As another example obviously supporting Warburg's hypothesis, low levels of cytochrome c oxidase (COX) were shown in a colon cancer cell line and postulated to be caused by impaired function of p53 in these cells, since p53 seems to be a necessary factor for the expression of an essential assembly factor for COX, SCO2 [15]. Since this central genome surveillance mechanism is mutated in about 50% of all cancers, this link was highlighted as an important and general mechanism to shut down OXPHOS activity in tumor cells.

However, quite on the contrary, a high enzymatic activity of COX, measured *in situ*, and high levels of the mitochondrial marker protein TOMM20 have been shown in a large series of papers in the rapidly proliferating, Ki67^+^ cells of breast cancers by the group of Lisanti, Sotgia and coworkers (reviewed in [16]). They postulated a three-compartment system of malignant, rapidly proliferating cancer cells with high OXPHOS capacity, which oxidize lactate, in a symbiotic relation with low OXPHOS tumor cells as well as cancer associated fibroblasts which provide the lactate and other nutrients derived from glucose and glutamine. In this tumor micro-environment, differential expression of the monocarboxylate transporters MCT1 and MCT4 in these different cell types may assist in the directional transport of lactate from producers to consumers [17], reviewed in [18]. This model was recently extended to head and neck squamous cell carcinoma (HNSCC) by the same group, in which the malignant tumor cells are derived from the COX- and TOMM20-rich, highly proliferative basal cell layer of this stratified epithelium [19].

Given these different findings, we decided to quantitate in a cohort of patients with HNSCC: (i) ^18^F-FDG-uptake, reflecting glucose consumption of the entire solid tumor mass, (ii) levels of the β-F1-ATPase and the BEC index within tumor cells as well as (iii) levels of representative subunits of the ETC, i.e. NDUF-S1 (Complex I) and COX1 (Complex IV). Our results show that high ^18^F-FDG-uptake correlates with poor prognosis, however, quite surprisingly, with high levels of β-F1-ATPase and a high BEC index in these tumors. No correlation was found between ^18^F-FDG-uptake and levels of Hsp60, GAPDH or other OXPHOS subunits. Therefore, we here describe a completely new phenotype of metabolic adaptation of tumor cells.

Since there is also a large body of literature on the possible role of pathological mtDNA mutations inactivating the OXPHOS system in tumors, we also decided to fully sequence the mtDNA of some representative tumor samples. We found putatively malignant, OXPHOS inactivating mutations in high ^18^F-FDG-uptake tumors, however with no clear relation to levels of the corresponding OXPHOS protein, indicating that they have rather randomly accumulated.

## RESULTS

**Figure 1:**
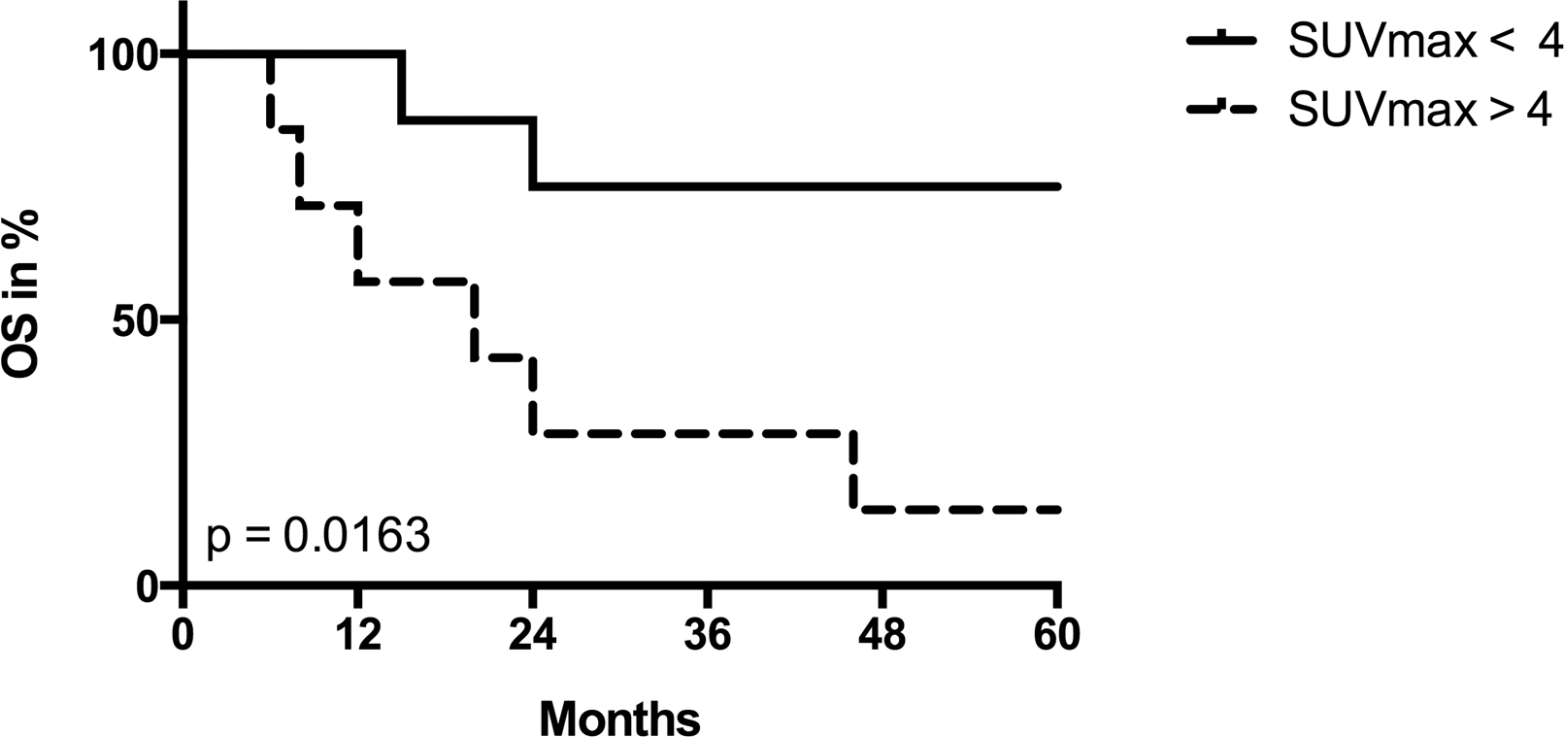
Univariate survival analysis stratified by SUV_max_ Kaplan-Meier plot for overall survival (OS) in patients with low (< 4) vs. high (> 4) SUV_max_ values. *P* value was derived by log-rank/Mantel-Cox test.

The clinical and pathological data of the HNSCC patients are given in Table 1. Since we intended to correlate quantitative data of mitochondrial protein levels in the tumor cells with FDG uptake of the solid tumor, we used a 60 min time point for determination of SUV_max_ in order to allow better discrimination between low and high uptake specimens. When we divided our patient cohort into two groups with high vs. low FDG uptake, overall survival after diagnosis was poor in patients with high SUV_max_ values (Figure 1).

**Table 1:** Characteristics of the analyzed head and neck cancer patients

*Clinicopathological feature*	*n*	*%*
**Total**	15	100
**Mean age**	61	
**Gender**		
Male	13	86.7
Female	2	13.3
**T classification**		
pT1	1	6.67
pT2	4	26.67
pT3	4	26.67
pT4	6	40.00
**N classification**		
pN0	6	40.00
pN1	1	6.67
pN2	6	40.00
pN3	2	13.33
**M classification**		
pM0	14	93.33
pM1	1	6.67
**Relapse**	4	26.66
**Death**	8	53.33
**HPV-status**		
Negative	6	40.00
Positive (Type 16)	9	60.00
**Localization**		
Cheek	1	6.67
Floor of mouth	2	13.33
Hypopharynx	2	13.33
Larynx	1	6.67
Oropharynx	7	46.67
Tongue	2	13.33

Summary of clinicopathological features of patients analyzed in this study. *n* = Number of patients.

**Figure 2:**
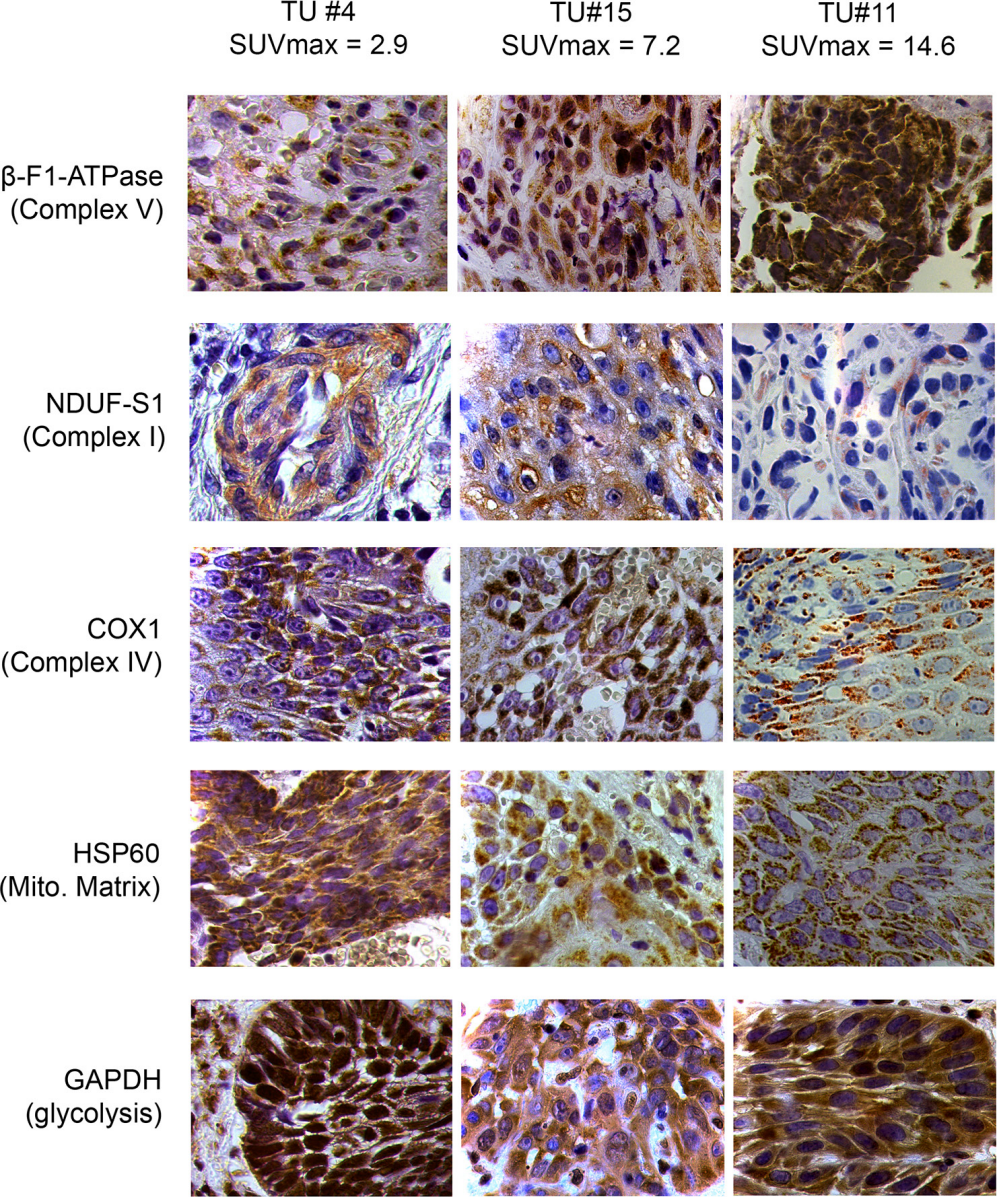
Representative immunohistochemical stainings of three HNSCCs for marker proteins quantified in this study Shown are images for tumors with low, intermediate and high SUVmax values (magnification x1000). Mito. Matrix = Mitochondrial Matrix, SUVmax = maximum standard uptake values.

**Figure 3:**
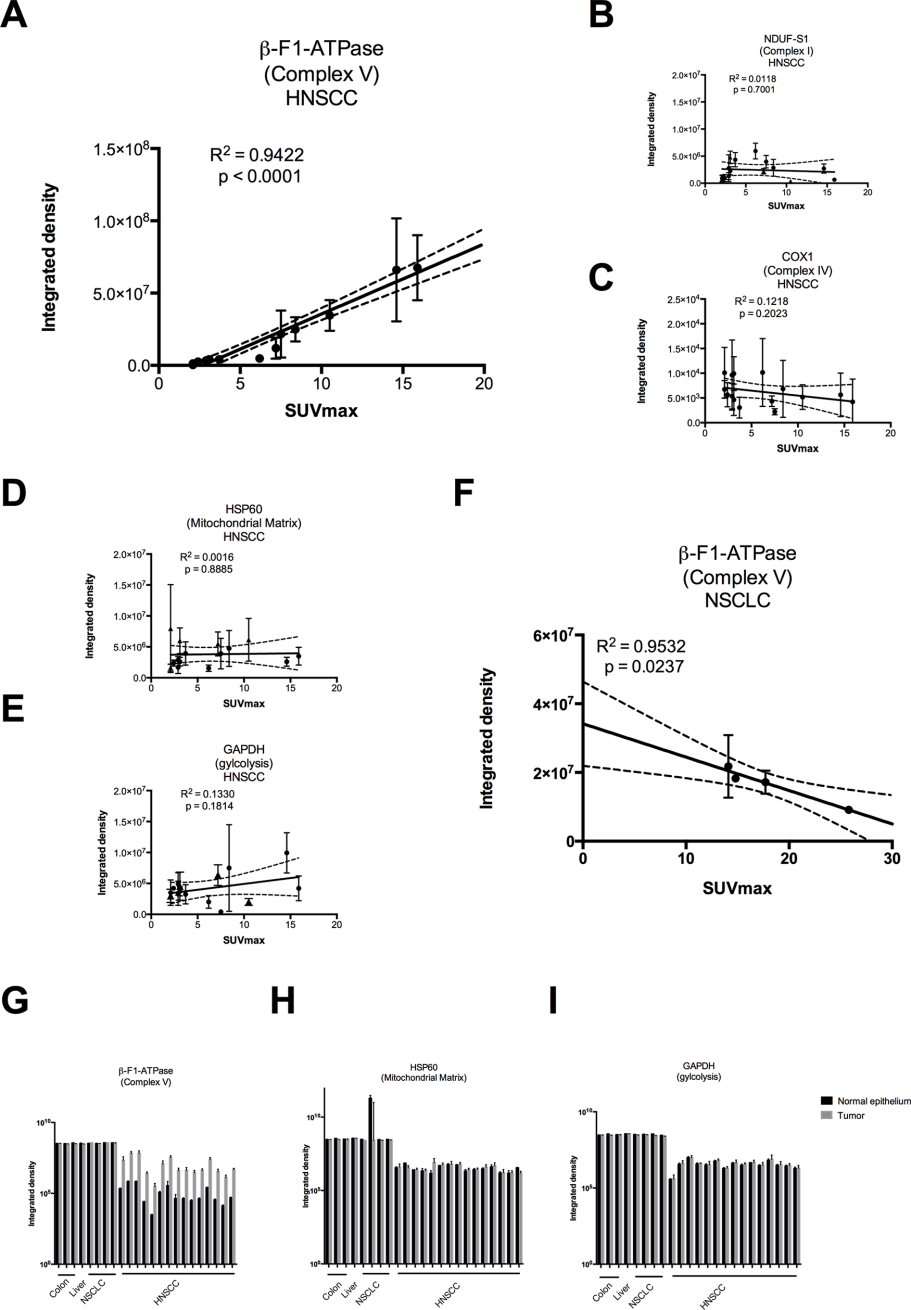
Regression analysis of staining intensities vs. SUV_max_ values A–F and raw densitometric data G–I in head and neck squamous cell carcinoma (HNSCC) vs. non-small cell lung carcinoma (NSCLC) Staining intensities were obtained by densitometric quantification of immunoreactions as shown in Figure 2 and are expressed in arbitrary units. SUVmax = maximum standard uptake values. (G – I) black bars = normal epithelium, grey bars = tumor. Please note the logarithmic scale.

**Figure 4:**
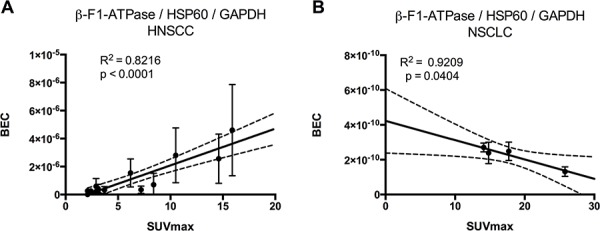
Regression analysis of BEC index [11] (β-F1-ATPase/Hsp60/GAPDH) vs. SUV_max_ BEC index = Bioenergetic cellular index, SUVmax = maximum standard uptake values. **A.** Head and neck carcinoma, **B.** non-small cell lung cancer.

Tumor samples were processed in a highly parallel way in order to be able to quantitate and compare staining intensities of the tumor cells in different samples. In addition, staining intensities of tumor cells were normalized to normal squamous epithelial cells to take into account any remaining intersample variability. Intensity of staining of the tumor cells was estimated in a first approach and results for β-F1-ATPase are shown in Supplementary Figure S1B. Representative examples from 3 patients covering the full range of obtained SUV_max_ values are displayed in Figure 2. Surprisingly, β-F1-ATPase levels were highest in tumor cells with high SUV_max_ values. This was confirmed by densitometric quantification of protein expression in all 15 tumor samples (Figure 3). We found a highly significant, direct correlation between β-F1-ATPase levels and SUV_max_ (Figure 3A), while levels of all other investigated proteins did not correlate at all (Figure 3B–3E). A highly significant correlation was also obtained when we related the BEC index (β-F1-ATPase/Hsp60/GAPDH, Figure 4A; [11] or levels of β-F1-ATPase/GAPDH (Supplementary Figure S1C) to SUV_max_ values. We excluded the possibility that a high SUV_max_ was due to a higher cell number per tumor volume, since this parameter was very similar in all samples (not shown). Our findings in HNSSC are in clear contrast to non-small cell lung carcinoma, where we were able to reproduce the inverse correlation between β-F1-ATPase levels and SUV_max_, as published before (Figure 3F and 4B) [11, 13]. Representative immunohistochemical stainings of non-small cell lung cancer are shown in Supplementary Figure S2. In addition, we directly compared protein expression between tumor and normal tissue from HNSCC, colorectal adenocarcinoma, hepatocellular carcinoma and non-small cell lung cancer (NSCLC; Figure 3G–3I). This revealed that β-F1-ATPase levels are generally higher in HNSCC tumors compared to the corresponding normal epithelium, but not in the other tumors analyzed (Figure 3G).

Inactivating mutations of p53 were postulated to impair complex IV assembly and thus to be a key mechanism for inactivation of the OXPHOS system in tumors carrying p53 mutations [15]. However, no relation was found between nuclear p53 enrichment, indicating an inactivating p53 mutation, and levels of COX 1 (Supplementary Figure S3A), an mtDNA encoded subunit which most likely represents the fully assembled complex [19] (and see below). All tumors which stained positive for p53 were found to be negative for HPV16, using p16^INK4A^ staining as a surrogate marker for HPV16 infection (Supplementary Figure S4) as well as nested PCR (not shown), thus there was also no correlation between SUV_max_ and HPV infection.

Finally, we fully sequenced the mtDNA of six tumors where we were able to collect by laser dissection samples containing > 75% of tumor cells. We found many polymorphisms compared to the revised Cambridge consensus sequence (GenBank RefSeq database: NC_012920; http://www.ncbi.nlm.nih.gov/nuccore/251831106; Supplementary Table S3), however all of them have been previously entered in the reference database as a normal variant of the highly polymorphic mtDNA [20]. In three tumors with low SUV_max_ values, we found a probably benign insertion in the TFAM binding site of the regulatory D-loop region (SUV_max_ 3.1), a missense mutation in the *COX1* gene (SUV_max_ 2.1), while one tumor had no mutation (SUV_max_ 2.9; Table 2). Two tumors with high SUV_max_ values carried a missense mutation in the ND1 subunit (SUV_max_ 6.2) and in the *Cyt b* gene (SUV_max_ 7.2), respectively, however with only 29% of the multi-copy mtDNA being mutated (heteroplasmy). In addition, one tumor (SUV_max_ 10.5) had two possibly pathogenic mutations in two different mitochondrial tRNA genes, which can be predicted to lead to an intra-mitochondrial translation defect, one in the homoplasmic and the other in the heteroplasmic state. Tumors with these mutations, together with their positions in the mtDNA sequence, are indicated in Supplementary Figure S3B, and Supplementary Table S3 gives references in which these mutations have been described, but controversially discussed regarding their possible pathogenicity.

**Table 2:** Mutations found in the mtDNA of 6 selected tumors

Patient Number	mtDNA-Position	Mutation	Comment
4	7080	T → C	association with prostate cancer [43] polymorphism in haplogroups U [44] and M12b [45]
5	524	C → CAC	found in haplogroups [45, 46]
12	4258	A → G	found in haplogroup M37 [20]
14	15045	G → A	found in haplogroup I1b [20]
15	5538	G → A	associated with late-onset encephalomyopathy [47]
	5628	T → C	CPEO / DEAF enhancer [48, 49] probably a neutral polymorphism [50] found in haplogroup L3e [20]

## DISCUSSION

This study was stimulated by the current discrepancies concerning Warburg-like metabolism in solid tumors: On the one hand, it is now clear that it is the need for building blocks as well as redox balance, and not the need for fuel, which drives high rates of glucose (as well as glutamine) uptake and release of lactate, respectively, even in the presence of oxygen [7, 21]. On the other hand, convincing data have been presented in a variety of tumors that indeed the OXPHOS system is impaired, specifically by low levels of the β-F1-ATPase subunit [22], and by high levels of the endogenous peptide inhibitor of the H^+^-F1-ATPase complex, IF-1 [23]. It is important to note here that there is general agreement in the field that levels of the subunits we measured here represent the fully assembled OXPHOS complexes, since unassembled subunits are present only in low amounts in the inner mitochondrial membrane and are very unstable [19]. A functional H^+^-F1-ATPase (complex V) is necessary for the execution of apoptosis, thus low levels have been suggested to be one of the many strategies tumor cells employ to escape cell death [10]. In addition, low levels of the H^+^-F1-ATPase, the main consumer of the proton-gradient, will lead to a high inner-membrane potential, which is commonly observed in many tumor cells [24], probably generating a reactive oxygen species (ROS) signal, which initiates important cellular stress responses [10, 25].

Surprisingly, in HNSCCs we found a completely different result, namely a striking positive correlation between levels of the β-F1-ATPase subunit and SUV_max_ values. Patients with tumors with high values had a poor prognosis (Figure 1). No correlation was found for the representative glycolytic enzyme GAPDH, and neither for the high abundance matrix protein Hsp60, probably reflecting mitochondrial mass, nor for two representative subunits of complex I and IV, respectively, of the OXPHOS system (Figure 3). Thus, HNSCCs seem to employ a completely different metabolic phenotype compared to the liver, kidney, colon, lung, gastric, ovarian and breast cancers which Cuezva and co-workers have extensively studied in the past and which we have confirmed in a small series of samples here (Figure 3 and Figure 4) (reviewed in[10]).

**Figure 5:**
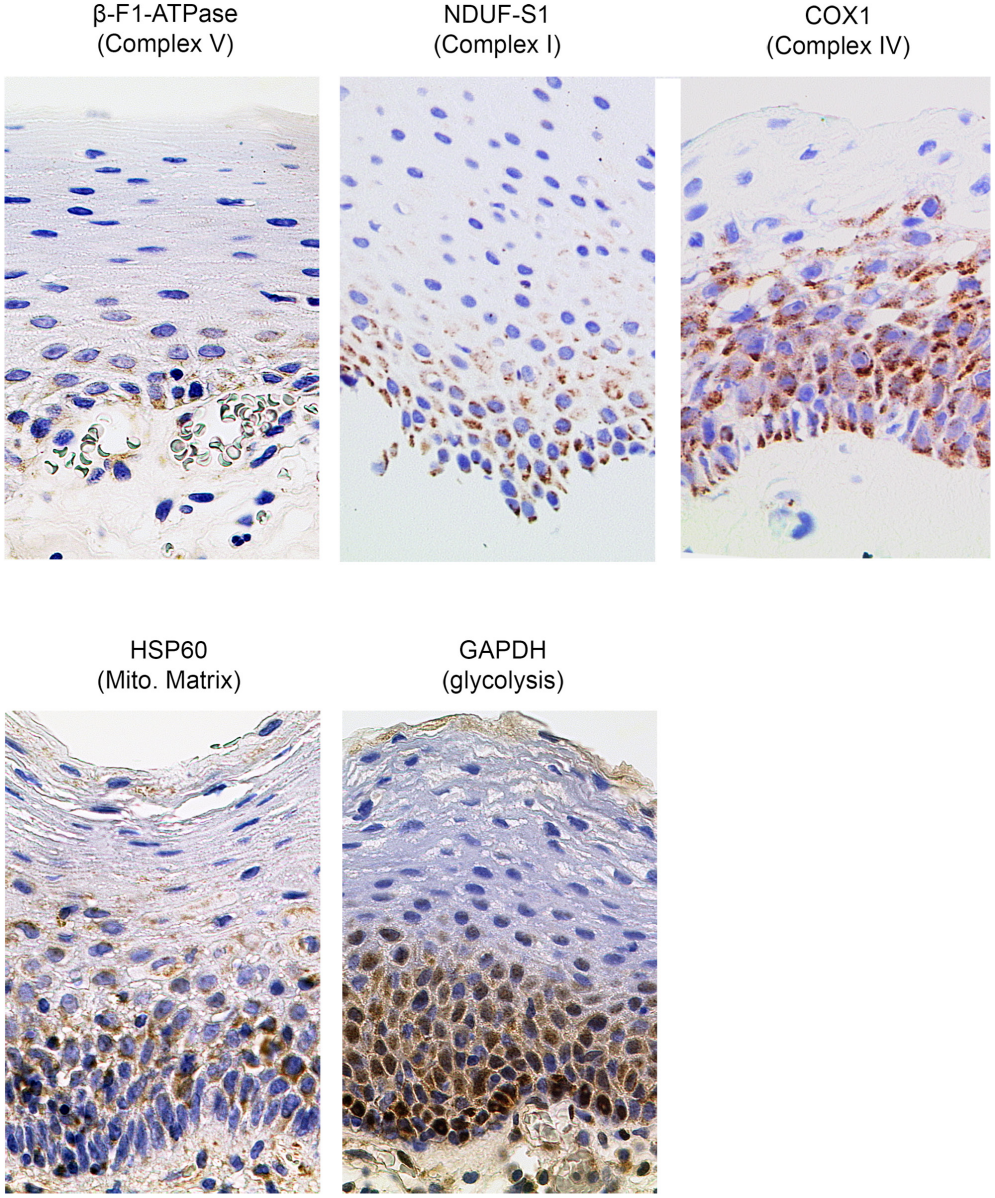
Immunohistochemical stainings in normal human mucosa for marker proteins quantified in this study (magnification x1000). Mito. Matrix = Mitochondrial Matrix

We propose the following explanation: The basal cell layer of squamous epithelia, from which HNSCCs are derived, is rather rich in mitochondria, compared to the underlying mesenchyme and the superficial layer (Figure 5). This was also recently shown for human mucosa and we had demonstrated this previously for human back skin epidermis [26]. This mitochondrial phenotype was kept during malignant transformation, since tumor cells stained much more intensely for all our mitochondrial marker proteins compared to the surrounding tumor stroma (Supplementary Figure S5). The representative glycolytic enzyme GAPDH is also highly expressed in the superficial layer (Figure 5), which is well separated from blood vessels, but also in transformed cells, indicating a high capacity for glycolytic flux in both cases, irrespective whether this may occur under anaerobic or aerobic conditions. In summary, both the highly proliferative basal layer as well as the highly proliferative tumor cells derived from it have a similar metabolic phenotype. When diverse healthy tissues are compared, the stoichiometry between different OXPHOS complexes can be different, however it remains rather stable in any given cell type under steady state conditions [27]. It is thus quite surprising to see an impressive 6fold increase of the H^+^-ATP synthase in high ^18^F-FDG-uptaking HNSCC, while other complexes as well as the mitochondrial mass marker HSP60 remain unchanged. We propose two models which may explain this finding: In one scenario, these highly proliferating malignant cells may indeed have a high demand for energy, which they take from nutrients provided by either blood or, indirectly, by surrounding stroma cells [28], thus indeed using the H^+^-ATP synthase for aerobic ATP synthesis. However we consider an alternative scenario more likely, keeping in mind that the basal cell layer is highly proliferative in the normal situation, too. When developing into a solid tumor, poorly perfused areas are formed due to an improper architecture of blood vessels, and this tumor hypoxia is reported to be especially a hallmark of HNSCCs [29, 30]. Under such conditions, ATP formed during glycolysis can be imported from the cytosol into the mitochondrial matrix, hydrolysed by the H^+^-ATP synthase running in reverse mode as ATPase, followed by export of ADP and inorganic phosphate, which can be converted into ATP again by glycolysis [31]. This on a first glance futile cycle generates a net charge difference across the inner mitochondrial membrane (negative inside), which, in the absence of electron flow to oxygen, serves to establish an inner membrane potential. This potential is absolutely necessary to import substrates and enzymes for essential mitochondrial synthetic functions like production of iron-sulfur clusters [32], membrane lipids [33] or heme [34], which are all building blocks needed for rapid cell proliferation. This pathway has been thoroughly investigated in cultured cells lacking mtDNA and, as a consequence, a functioning respiratory chain (ρ0-cells; [31]), and we could show that it is sufficient to allow normal proliferation of ρ0-cells [35]. When we completely ablated mtDNA *in vivo* using K14-Cre mediated knock-out of the maintenance factor mitochondrial transcription factor A (TFAM), the affected keratinocytes formed a normal epidermis in mice and even hyperproliferated, thus proving that this pathway indeed can operate *in vivo* [36].

In addition, based on our results, we can exclude a selective downregulation of cytochrome c oxidase (complex IV) in HNSCC tumors carrying an inactivating p53 mutation, which was postulated based on results from cultured colon carcinoma cells [15], since nuclear enrichment of this major tumor suppressor was found in samples with high COX1 levels (Supplementary Figure S3A). Finally, we show here that the accumulation of mtDNA mutations, as another possible mechanism to inactivate the OXPHOS system, is probably not important and is therefore rather a stochastic process caused by random drift in the rapidly proliferating tumor cells, as shown previously [37]. For example, the tumor with SUV 6.2 containing the m.4258A > G mutation in ND1, which leads to a putatively missense Thr to Ala exchange, has the highest complex I levels we observed, and the tumor with a SUV of 2.1 with a m.7080T > C mutation, leading to a putatively missense Phe to Leu exchange in COX1 has the highest COX 1 levels (Supplementary Figure S3B). In contrast, the tumor with a very high SUV of 10.5 carries two potentially harmful mutations in two mitochondrial tRNAs, has average COX1 levels (Supplementary Figure S3B) and very low levels of NDUF-S1 (complex I, Figure 3B), a situation typical for cells carrying inactivating mitochondrial tRNA mutations [38, 39], since complex I has seven mtDNA encoded subunits. High levels of β-F1-ATPase in this tumor indeed do not exclude the presence of a mitochondrial translation defect caused by these mutations, since as the exception to the rule, a truncated, non-functional ATPase containing β-F1-ATPase can indeed be assembled even in the absence of the two mtDNA encoded complex V subunits. In conclusion, we found putatively malignant, inactivating mtDNA mutations, however with no clear relation to SUV_max_ values or levels of the corresponding OXPHOS proteins, indicating that they have randomly accumulated by clonal expansion of founder mutations in the tumor initiating cells and do not cause an inactivation of the OXPHOS system.

In summary, we here show a new metabolic phenotype employed by HNSCCs, derived from the mitochondria-rich basal layer of the mucosa, which probably enables those tumors with highest levels of β-F1-ATPase and complex V of the ETC to rapidly proliferate even in hypoxic zones, which are typical for these solid tumors. Patients with high complex V containing tumors have the poorest prognosis, thus they may profit from treatment with complex V inhibitors.

## PATIENTS, MATERIALS AND METHODS

### Subjects and material

Fresh frozen and formalin-fixed, paraffin embedded HNSCC samples from 15 patients treated at the Department of Otorhinolaryngology and Head and Neck Surgery of the University Hospital of Köln, Germany, between 2005 and 2008 were obtained at the time of surgical resection. Inclusion criteria were the availability of sufficient fresh frozen tumor tissue containing ≥ 70% tumor cells and the availability of dynamic ^18^F-FDG-PET data. Table 1 summarizes the clinicopathological features of the HNSCC cases used in this study. Together with additional cases from colorectal adenocarcinoma (*n* = 3), hepatocellular carcinoma (*n* = 1), and non-small cell lung cancer (*n* = 4), all samples were collected from the archive of the Department of Pathology, University Hospital of Köln, Germany.

### Ethics statement

Patient material was used according to the code for proper secondary use of human tissue. The ethics committee of the Medical Faculty of the University of Köln approved this study. Written, informed consent had been obtained from all patients.

### ^18^F-fluorodeoxyglucose positron emission tomography

All patients were scanned by ^18^F-FDG-PET (ECAT EXACT HR Scanner model 921, Siemens-CTI, Knoxville, TN, USA) at the Department of Nuclear Medicine, University of Köln, Germany. After fasting for at least 6 hours, patients underwent intravenous injection of 370 MBq ^18^FDG. Image acquisition was performed over a time course of 60 minutes with an interval of 3 min between each image for the first 30 min and a last image after 60 min (dynamic PET). Standard Uptake Values (SUV) of ^18^FDG were analyzed semiquantitatively by defining a ‘Region of Interest’ (ROI) of the tumors spanning 10 pixels with the highest intensity and the values for the 60 min time point was used to define SUV_max_.

### Immunohistochemical staining

Four μm sections were cut from paraffin embedded, formalin-fixed tissue blocks, mounted on silane coated slides, dried overnight and deparaffinised by routine techniques in xylene and rehydrated in ethanol (100%, 100%, 90%, 70%, 50%). Sections were then washed in Tris-HCl buffer (pH 8.0) before incubation with primary antibodies. Antibodies used in this study are given in Table 3. All incubations were performed for 0.5 h at room temperature. Tumor samples used for quantification were stained simultaneously with each antibody in order to guarantee same conditions for later quantification. Labeling was detected semiautomatically by DAKO TechMate™ 500 Plus with the DAKO REAL™ Detection System using the streptavidine-biotin system according to manufacturer's protocol (Dako REAL™ Biotinylated Secondary Antibodies: goat anti-mouse and anti-rabbit immunoglobulins, Dako REAL™ streptavidine peroxidase, Dako REAL™ AEC/H2O2 Substrate Solution, Dako REAL™ Blocking Solution, Dako REAL™ Buffer Kit). Sections were washed (3 times 5 min in PBS), counterstained with haematoxylin and finally mounted in Aquatex (Merck, Darmstadt, Germany).

**Table 3:** Antibodies used in this study

Antibody	Animal source	Dilution	Pretreatment	Vendor
OXPHOS Complex IV subunit COX 1	mouse monoclonal	1:1000	none	Invitrogen Corporation
Hsp60	rabbit polyclonal	1:1000	none	Abcam, ab46798, lot. GR51423
OXPHOS Complex V subunit ATP5B	rabbit polyclonal	1:500	citrate puffer (pH 6.0)	Sigma, AV48185, lot.QC18082
GAPDH	mouse monoclonal	1:1000	citrate puffer (pH 6.0)	Abcam, ab8245, lot.GR1883
OXPHOS Complex I subunit NDUF-S1	rabbit polyclonal	1:1000	none	Sigma, N6039
p16INK4A surrogate marker for HPV-driven carcinogenesis	mouse monoclonal	1:50	none	BD Biosciences, clone G175–405
p53	mouse monoclonal	1:25	none	BioLogo, clone DO-7

### Quantification of protein expression

The immunostaining results for the OXPHOS complexes I, IV and V, HSP60 and GAPDH were scored independently by two investigators using different approaches: (a) an expert pathologist (ACA) quantified the staining intensity of tumor cells in comparison to normal squamous epithelium on the same sample (1, weak; 2, moderate; and 3, strong overexpression in the tumor cells); and (b) by densitometric measuring of the stain deposition in the cytoplasm of tumor and normal epithelial cells (CUH). For this purpose, up to 10 digital pictures of tumor regions of each sample (minimum 7 because of tumor size limitations) and 3 digital pictures of normal squamous epithelium of each sample were taken at 1000-fold magnification with a Zeiss Axiocam MRc camera (Carl Zeiss, Oberkochen, Germany). The cytoplasm of all tumor cells or normal epithelial cells of each picture was manually marked as ‘Region Of Interest’ (ROI) using the ImageJ 1.47 program and the integrated density values of the selected regions were calculated [40]. Subsequently, the mean integrated density values of all images of each sample were calculated and subtracted by the mean integrated density values of normal cytoplasm of epithelial cells from the same sample. Error bars were calculated using the Gaussian error propagation law.

### Laser-capture microdissection of tumor tissue for extraction of DNA and mtDNA sequencing

For sequencing of mtDNA, frozen sections were mounted on ultraviolet irradiated PALM membrane glass slides (Micro Laser Biotechnologies, Bernried, Germany). Subsequently sections were shortly dried. The areas of interest containing at least 75% of tumor cells were cut out using the PALM microdissection system and collected in 100 μl-tube caps containing 10 μl lysis buffer (20 mM Tris-HCl pH 8.0, 1 mM EDTA, 0.2 mg / ml proteinase K, 0.01% Tween). After centrifugation (6000 g, 3 min) the obtained fragments were incubated in lysis buffer for at least 3 hours at 65°C. Proteinase K was inactivated for 5 min at 95°C. Remaining particles were sedimented (13000 rpm, 10 min) and the supernatant was used for further analysis steps. After extraction of total DNA, samples were split for duplicates into two parts. From each sample, four overlapping 4kb fragments were amplified (primers: R2/R37, R9/R43, R16/R49, R23/R30; see Supplementary Table S1) covering the whole mtDNA. In the second, nested PCR reaction, 400–600 bp long fragments were generated for the sequencing reactions (Primer combinations see Supplementary Table S2). For all PCR reactions, the Expand Long polymerase (Roche, Mannheim, Germany) was used according to manufacturer's instructions. Polymerase chain reaction products were sequenced by MWG Operon (Ebersberg, Germany). Sequences were compared with the revised Cambridge consensus sequence (GenBank RefSeq database: NC_012920; http://www.ncbi.nlm.nih.gov/nuccore/251831106) and the MITOMAP database (http://www.mitomap.org) was used for determining if the detected mutations were regarded as pathologic. Sequence results were analyzed using the BLASTN program.

### DNA isolation, and HPV typing by PCR

For analysis of HPV16 infection, DNA was isolated from fresh frozen tumor samples using the QIAamp Tissue Kit (Qiagen, Hilden, Germany) according to the manufacturer's instructions. Total cellular DNA was eluted with 250 μl of the AE-buffer (Qiagen) and 5 μl were used in each of the PCR analyses.

To test the quantity and quality of the DNA samples and to demonstrate that the samples were free from inhibitory substances, PCR was performed for the β-Globin gene, resulting in a 268 bp PCO4/GH20 PCR product [41].

HPV sequences were detected in samples from the entire tumor by highly sensitive group-specific nested PCR protocols with degenerate primers A5/A10 and A6/A8 (Table 2) for HPV as previously described [42]. PCR products were sequenced (GATC Biotech, Konstanz, Germany) and sequencing results were analyzed using the BLASTN program.

### Statistics

All statistical analyses were performed using GraphPad Prism 6.0 (GraphPad Software, La Jolla California, USA) and a significance level of *p* < 0.05 was chosen for all analyses. An association between expression levels of the markers in cancerous tissue normalized to expression in normal tissues and SUV_max_ was analyzed using Student's *t*-test. Overall survival rates were estimated for a time period of 5 years using the Kaplan–Meier algorithm for incomplete observations. For practical and statistical purposes as based on the results, a dichotomous grouping was done, defining a SUVmax between 0–4 as low and > 4 as high. The overall survival time was defined as the interval between the date of diagnosis and the last date when the patient was known to be alive (censored) or date of death for any reason (uncensored). Univariate analysis of the various variables was performed with the log-rank test.

## SUPPLEMENTARY FIGURES AND TABLES


