# Supplementary material for: High glucose uptake unexpectedly is accompanied by high levels of the mitochondrial β-F1-ATPase subunit in head and neck squamous cell carcinoma
Source: Oncotarget. 2015 Oct 6;6(34):36172–84. doi: 10.18632/oncotarget.5459 (PMC4742169; doi:10.18632/oncotarget.5459)
Supplement: Supplementary file 1 [file oncotarget-06-36172-s001.pdf]

SUPPLEMENTARY FIGURES AND TABLES

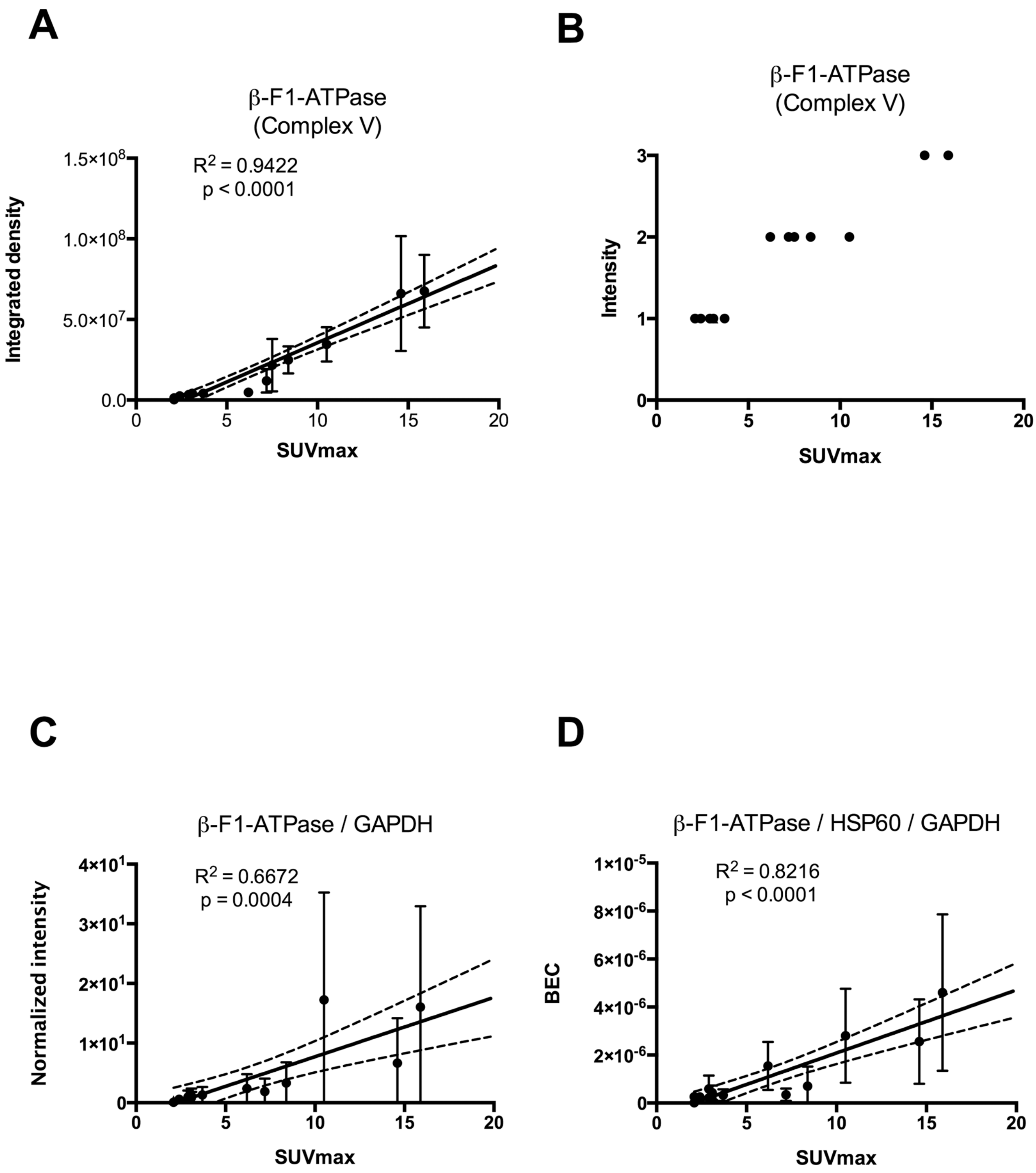

Supplementary Figure S1: Overview of β-F1-ATPase staining intensities without (A, B) and with normalization to other proteins (C, D; arbitrary units) in HNSCCs. Graphs (A) and (D) have been already shown as regular Figures (Figure 3) and are repeated here for better comparison **A.** Regression analysis of β-F1-ATPase staining intensity vs. SUVmax. **B.** Semi-quantification of β-F1-ATPase staining intensity vs. SUVmax as assessed by a trained pathologist. **C.** Normalization of β-F1-ATPase staining intensity to GAPDH. **D.** Regression analysis of BEC index vs. SUVmax. BEC index = Bioenergetic cellular index, SUVmax = maximum standard uptake values.

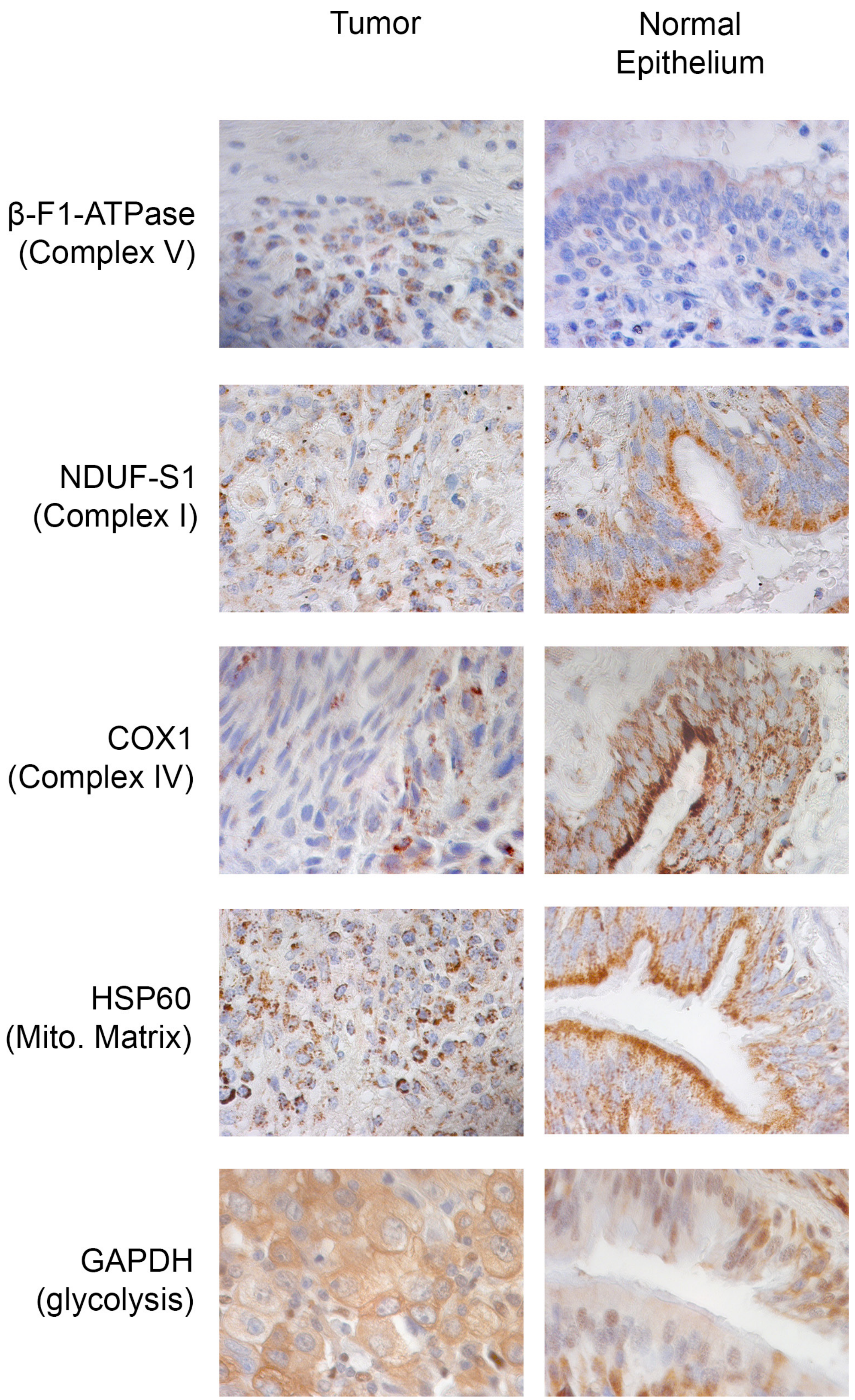

Supplementary Figure S2: Representative immunohistochemical stainings of non-small cell lung cancer (NSCLC) (magnification x1000). Mito. Matrix = Mitochondrial matrix.

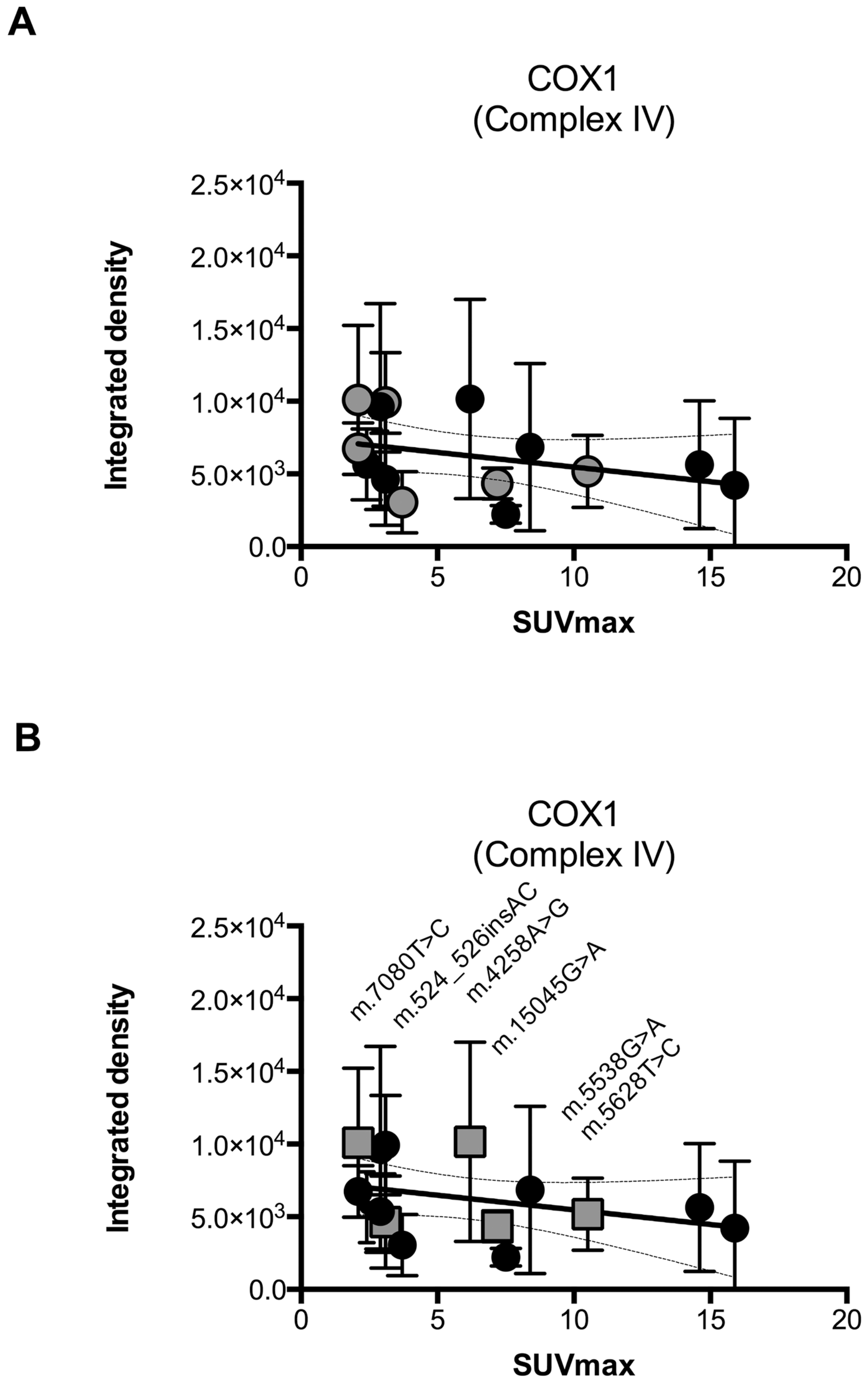

Supplementary Figure S3: Regression analysis of staining intensities for COX 1 subunit of complex IV vs. SUVmax, as shown in Figure 3 in HNSCCs. A. Cases with p53 positive nuclear immunostaining, which are at the same time negative for HPV16 infection, are indicated by grey circles. **B.** Tumors with mtDNA mutations and their positions in the sequence are indicated by a rectangle. SUVmax = maximum standard uptake values.

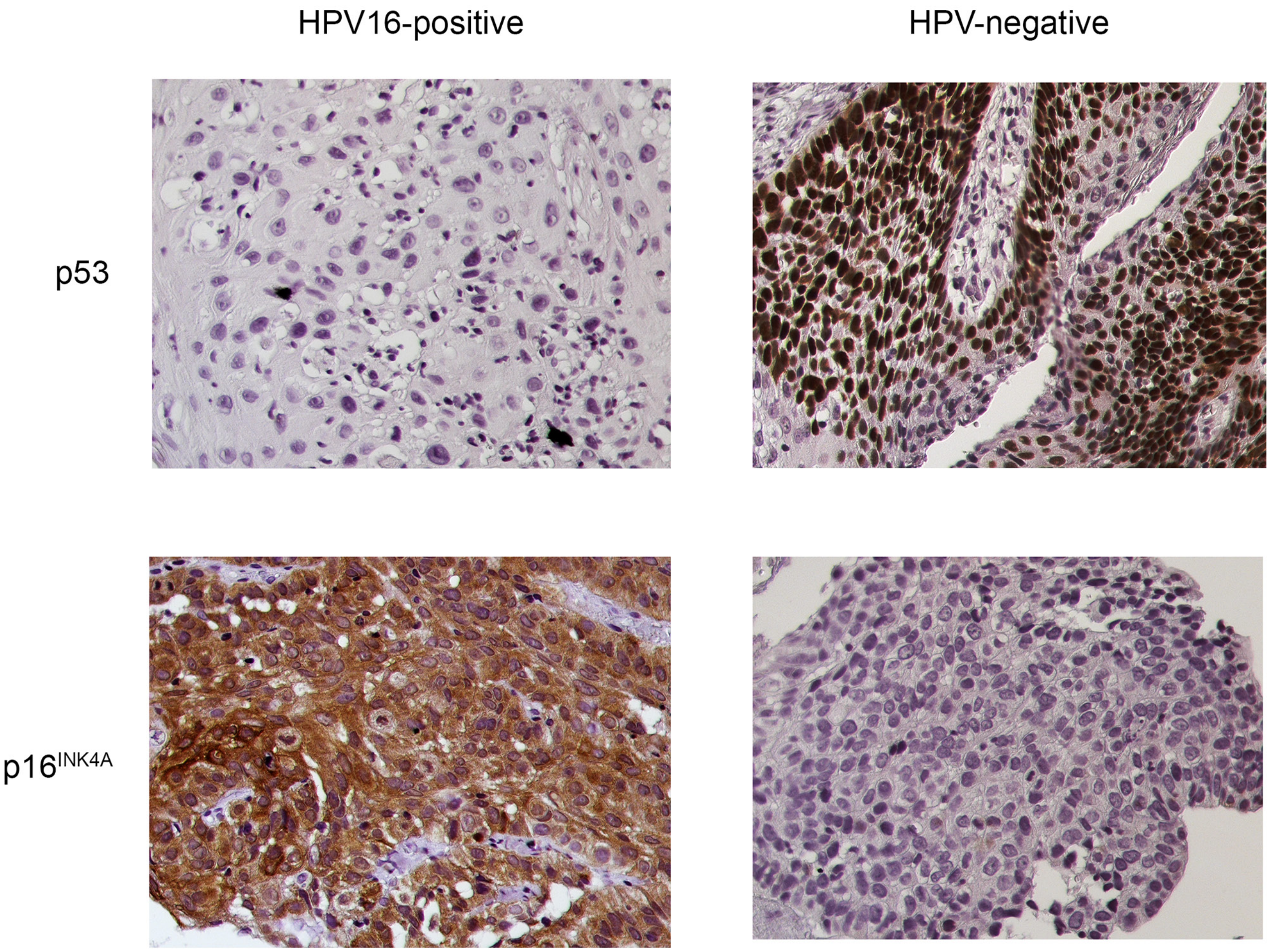

Supplementary Figure S4: Immunohistochemical staining demonstrating absence of p53 positive nuclei in HPV16-positive HNSCCs, which are positive for p16^{INK4A} as surrogate marker (magnification x400).

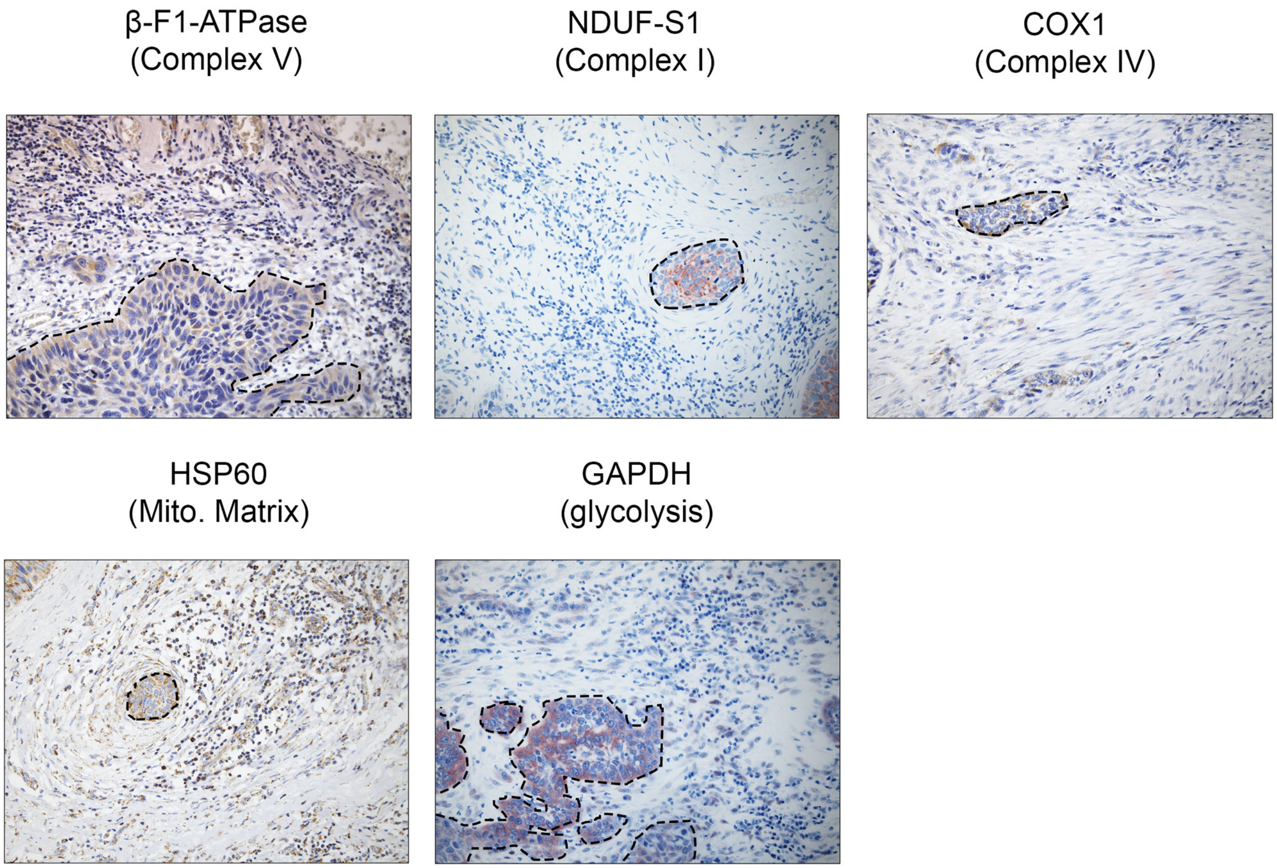

Supplementary Figure S5: Immunohistochemical stainings demonstrating differences between tumor cells and tumor stroma (magnification x400) in HNSCCs. Tumor regions are marked by black dotted lines. Mito. Matrix = Mitochondrial Matrix.

Supplementary Table S1: Primer sequences used for PCR amplification of the mitochondrial genome (R) as well as HPV16 sequences (A).

Primer name	Sequence
R1	ACTCCACCATTAGCACCCAAAGC
R2	CATAAAGCCTAAATAGCCCACACG
R3	ACACCGCTGCTAACCCCATAC
R4	AGCTAAGACCCAAACTGGGATT
R5	AGTGCACTTGGACGAACCAGAG
R6	CAAAGAGGAACAGCTCTTTGGA
R7	ACCTGTATGAATGGCTCCAC
R8	GAGAAATAAGGCCTACTTCACAAAGC
R9	GAACTAGTCTCAGGCTTCAACATCG
R10	CGGACAATGAACCATAACCAATACTA
R11	CCTTACCACGCTACTCCTACCTATCTCC
R12	CAATTCAATATGAAAATCACCTCGG
R13	CTTAGGGGCCATCAATTTCATCAC
R14	CCCACTTCCACTATGTCCTATCAAT
R15	CACATTCGAAGAACCCGTATACATAAA
R16	CCACAGTTTCATGCCCATCGTCC
R17	CCCCTCTAGAGCCCACTGTA
R18	CTGTCGCCTTAATCCAAGCCTACG
R19	GCCTGATACTGGCATTTTGT
R20	CACCTCATATCCTCCCTACTATGC
R21	GCTCCCTTCCCCTACTCATC
R22	CACGTTCTCCTGATCAAATATCAC
R23	TCATCCCTGTAGCATTGTTCG
R24	CTTCCACCCCCTAGCAGAA
R25	TCGCTACCTCCCTGACAAGC
R26	CCTCAACCCAAAAAGGCATAA
R27	CCCCCATAAATAGGAGAAGGCTTA
R28	CCTGAAACATCGGCATTATCC
R29	CCACATCAAGCCCGAATGA
R30	TGGAGAGCTCCCGTGAGTGGTT
R31	GGTGATGTGAGCCCGTCTAAAC
R32	AGTGTTCTGGCGAGCAGTTTTG
R33	GGTTTAGCTCAGAGCGGTCAAG
R34	GGGTCTTCTCGTCTTGCTGTGT
R35	GGCTCTGCCATCTTAACAAACC
R36	CTAGGAAGATTGTAGTGGTGAGGGTG

(*Continued*)

Primer name	Sequence
R37	TGCCTTGGGTAACCTCTGGGACTC
R38	TTGCGTTCAGTTGATGCAGAGTGG
R39	CTCCAGCTCATGCGCCGAATAATAG
R40	TAGGACTGCTGTGATTAGGACGG
R41	TGGATTTTGGCGTAGGTTTGGTCT
R42	GGAAAATGATTATGAGGGCGTGAT
R43	CGGTAGTATTTAGTTGGGGCATTTCAC
R44	GATAGGCATGTGATTGGTGGG
R45	GAATGTTGTCAAAACTAGTTAATTGGAAGT
R46	GGCGGCAAAGACTAGTATGG
R47	TTCTTGGGCAGTGAGAGTGA
R48	CCCCATTGTGTTGTGGTAAA
R49	GAAGAACTGATTAATGTTTGGGTCT
R50	GTGGTGATAGCGCCTAAGCA
R51	GGGTGGGGTTATTTTCGTTAATGT
R52	GAGATTGCTCGGGGGAATAG
R53	GGTTGTAGTCCGTGCGAGAA
R54	ACTGTGGCCCCTCAGAATGA
R55	CAAGGACGCCTCCTAGTTTG
R56	CGGTTGTTGATGGGTGAGT
A5	TATTYTSCTWCTCCYAGTGG
A6	GCMCAGGGMCAYAAYAATGG
A8	CAAARTTCCARTCYTCCAA
A10	CKTCCCAARGGAWAYTGRTC

Supplementary Table S2: Combination of primers used for amplification of the mitochondrial genome.

Primer names are indicated according to Table 2. Fragment length in base pairs (bp). Positions in the mitochondrial genome are given according to the revised Cambridge consensus sequence (GenBank RefSeq database: NC_012920; http://www.ncbi.nlm.nih.gov/nuccore/251831106).

Primer pairs	Fragment length (bp)	Section of mt-DNA
1: R1/R30	640	15974 - 45
2: R2/R31	687	16520 - 638
3: R3/R32	623	521 - 1144
4: R4/R33	622	1057 - 1679
5: R6/R34	628	2101 – 2729
6: R7/R35	624	2622 – 3246
7: R8/R36	899	3137- 4036
8: R9/R37	911	3921 – 4832
9: R10/R38	913	4712 – 5625
10: R11/R39	523	5462 – 5985
11: R12/R40	687	5798 – 6485
12: R13/R41	764	6377 – 7141
13: R14/ R42	655	7027 – 7682
14: R15/ R43	973	7413 – 8386
15: R16/R44	1037	8194 - 9231
16: R18/R45	907	9140 - 10047
17: R19/R46	752	9921 – 10673
18: R20/R47	772	10542 – 11314
19: R21/R48	783	11226 – 12009
20: R22/R49	779	11911 – 12690
21: R23/R50	588	12602 - 13190
22: R24/R51	563	13122 - 13685
23: R25/R52	592	13577 – 14169
24: R26/R53	629	14066 - 14695
25: R27/R54	592	14588 -15180
26: R28/R55	542	15081 - 15623
27: R29/R56	540	15544 - 16084

Supplementary Table S3: mtDNA polymorphisms found in 6 tumor samples, compared to the revised reference sequence.
(GenBank RefSeq database: NC_012920; http://www.ncbi.nlm.nih.gov/nuccore/251831106).

patient	mtDNA-position	polymorphism	homo-plasmy	AA-change	locus
**1**	73	A → G	100%		Control region
	263	A → G	100%		Control region
	315	C → CC	100%		Control region
	497	C → T	100%		Control region
	1189	T → C	100%		Noncoding
	1438	A → G	100%		Noncoding
	2706	A → G	100%		Noncoding
	3480	A → G	100%	Syn	NADH/1
	4295	A → G	100%		Noncoding
	4769	A → G	100%	Syn	NADH/2
	6260	G → A	100%	Syn	Cox/1
	7028	C → T	100%	Syn	Cox /1
	8860	A → G	100%	Threonine → Alanine	ATP-Synthase/6
	9377	A → G	100%	Syn	Cox/3
	9698	T → C	100%	Syn	Cox/3
	10398	A → G	100%	Threonine → Alanine	NADH/3
	10550	A → G	100%	Syn	NADH/4L
	11251	A → G	100%	Syn	NADH/4
	11467	A → G	100%	Syn	NADH/4
	11485	T → C	100%	Syn	NADH/4
	11719	G → A	100%	Syn	NADH/4
	11840	C → T	100%	Syn	NADH/4
	12308	A → G	100%		Noncoding
	12372	G → A	100%	Syn	NADH/5
	13740	T → C	100%	Syn	NADH/5
	14167	C → T	100%	Syn	NADH/6
	14766	C → T	100%	Threonine → Isoleucine	Cytochrom b
	14798	T → C	100%		Cytochrom b
	15326	A → G	100%	Syn	Cytochrom b
	15884	G → A	100%	Alanine → GlycineAlanine → Threonine	Cytochrom b
	16224	T → C	100%		Control region
	16245	C → T	100%		Control region

(*Continued*)

patient	mtDNA-position	polymorphism	homo-plasmy	AA-change	locus
	16311	T → C	100%		Control region
**2**	119	T → C	100%		Control region
	189	A → G	100%		Control region
	195	T → C	100%		Control region
	204	T → C	100%		Control region
	207	G → A	100%		Control region
	263	A → G	100%		Control region
	315	C → CC C →	100%		Control region
	709	CCG → A	100%		Noncoding
	750	A → G	100%		Noncoding
	1243	T → C	100%		Noncoding
	1438	A → G	100%		Noncoding
	2706	A → G	100%		Noncoding
	3505	A → G	100%	Threonine → Alanine	NADH/1
	4769	A → G	100%	Syn	NADH/2
	5046	G → A	100%	Valine → Isoleucine	NADH/2
	5460	G → A	100%	Alanine → Threonine	NADH/2
	5580	T → C	100%		Noncoding
	7028	C → T	100%	Syn	Cox/1
	7864	C → T	100%	Syn	Cox/2
	8860	A → G	100%	Threonine → Alanine	ATP-Synthase/ 6
	8994	G → A	100%	Syn	ATP-Synthase/6
	11674	C → T	100%	Syn	NADH/4
	11719	G → A	100%	Syn	NADH/4
	11947	A → G	100%	Syn	NADH/4
	12414	T → C	100%	Syn	NADH/5
	12705	C → T	100%	Syn	NADH/5
	14766	C → T	100%	Threonine → Isoleucine	Cytochrom b
	15326	A → G	100%	Alanine → Glycine	Cytochrom b
	15884	G → C	100%	Alanine → Threonine	Cytochrom b
	16158	A → G	22%		Control region
	16292	C → T	100%		Control region
	16519	T → C	100%		Control region
**6**	73	A → G	100%		Control region
	152	T → C	100%		Control region
	217	T → C	100%		Control region

(*Continued*)

patient	mtDNA-position	polymorphism	homo-plasmy	AA-change	locus
	263	A → G	100%		Control region
	310	T → C	100%		Control region
	340	C → T	100%		Control region
	508	A → G	100%		Control region
	750	A → G	100%		Noncoding
	1438	A → G	100%		Noncoding
	2706	A → G	100%		Noncoding
	3116	C → T	100%		Noncoding
	3720	A → G	100%	Syn	NADH/1
	4769	A → G	100%	Syn	NADH/2
	5390	A → G	100%	Syn	NADH/2
	5426	T → C	100%	Syn	NADH/2
	6045	C → T	100%	Syn	Cox/1
	6152	T → C	100%	Syn	Cox/1
	7028	C → T	100%	Syn	Noncoding
	8860	A → G	100%	Threonine → Alanine	ATP-Synthase/6
	10876	A → G	100%	Syn	NADH/4
	11197	C → T	100%	Syn	NADH/4
	11467	A → G	100%	Syn	NADH/4
	11719	G → A	100%	Syn	NADH/4
	11732	T → C	100%	Syn	NADH/4
	12308	A → G	100%		Noncoding
	12372	G → A	100%	Syn	NADH/5
	13020	T → C	100%	Syn	NADH/5
	13734	T → C	100%	Syn	NADH/5
	14766	C → T	100%	Threonine → Isoleucine	Cytochrom b
	15326	A → G	100%	Alanine → Glycine	Cytochrom b
	15907	A → G	100%		Noncoding
	16051	A → G	100%		Control region
**8**	73	A → G	100%		Control region
	263	A → G	100%		Control region
	315	C → CC C → T	100%		Control region
	497	A → G	100%		Control region
	750	T → C	100%		Noncoding
	1189	A → G	100%		Noncoding
	1438	A → G	100%		Noncoding

(*Continued*)

patient	mtDNA-position	polymorphism	homo-plasmy	AA-change	locus
	2706	A → G	100%		Noncoding
	3480	T → C	100%	Syn	NADH/1
	3552	A → G	100%	Syn	NADH/2
	4769	A → G	100%	Syn	Cox/1
	6060	C → CC A → G	100%	Isoleucine → Valine	Cox/1
	7028	T → C	100%	Syn	Noncoding
	8860	A → G	100%	Threonine → Alanine	ATP-Synthase6
	9698	T → C	100%	Syn	Cox/3
	11251	A → G	100%	Syn	NADH/4
	11299	G → A	100%	Syn	NADH/4
	11467	G → A	100%	Syn	NADH/4
	11719	A → G	100%	Syn	NADH/4
	11914	G → A	100%	Syn	NADH/4
	12308	C → T	61%		Noncoding
	12372	C → T	73%	Syn	NADH/5
	14167	T → C	100%	Syn	NADH/6
	14766	A → G	100%	Threonine → Isoleucine	Cytochrom b
	14798	G → A	100%		Cytochrom b
	15799	T → C	100%	Syn	Cytochrom b
	16129	T → C	100%	Syn	Control region
	16224	T → C	100%		Control region
	16311		100%		Control region
	16519		100%		Control region
**11**	709	G → A	100%		Noncoding
	750	A → G	100%		Noncoding
	1438	A → G	100%		Noncoding
	2706	A → G	100%		Noncoding
	2850	T → C	100%		Noncoding
	4216	T → C	100%	Tyrosine → Histidine	NADH/1
	4769	A → G	100%	Syn	NADH/2
	4917	A → G	100%	Asparagine → Aspartate	NADH/2
	4931	C → T	100%		NADH/2
	7022	T → C	100%	Syn,	Cox/1
	7028	C → T	100%	Syn	Cox/1
	7702	G → A	45%	Syn	Cox/2

(*Continued*)

patient	mtDNA-position	polymorphism	homo-plasmy	AA-change	locus
	8697	G → A	100%	Syn	ATP-Synthase/6
	8860	A → G	100%	Syn	ATP-Synthase/6
	9545	A → G	100%	Threonine → Alanine	Cox/3
	10463	T → C	100%	Syn	Noncoding
	11251	A → G	100%		NADH/4
	11719	G → A	100%	Syn	NADH/4
	11812	A → G	100%	Syn	NADH/4
	13368	G → A	100%	Syn	NADH/5
	13965	T → C	100%	Syn	NADH/5
	14233	A → G	100%	Syn	NADH/6
	14766	C → T	100%	Syn	Cytochrome b
	14905	G → A	100%	Threonine → Isoleucine	Cytochrome b
	15326	A → G	100%	Syn	Cytochrome b
	15452	C → A	100%	Alanine → Glycine	Cytochrome b
	15928	G → A	100%	Leucine → Isoleucine	Noncoding
	16126	T → C	100%		Control region
	16294	C → T	100%		Control region
	16519	T → C	100%		Control region
**16**	43	C → CC A → G	100%		Control region
	750	A → G	100%		Noncoding
	1438	T → C	100%		Noncoding
	3866	A → G	100%	Isoleucine Threonine	NADH/1
	4769	A → G	100%	Syn	NADH/2
	8860	C → T	100%	Threonine → Alanine	ATP-Synthase/6
	11674	G → A	100%	Syn	NADH/4
	11719	A → G	100%	Syn	NADH/4
	11947	A → G	100%	Syn	NADH/4
	15326	C → T	100%	Alanine → Glycine	Cytochrome b
	16261	T → C	100%		Control region
	16519		100%		Control region
